# Elucidation of the viral disassembly switch of tobacco mosaic virus

**DOI:** 10.15252/embr.201948451

**Published:** 2019-09-19

**Authors:** Felix Weis, Maximilian Beckers, Iris von der Hocht, Carsten Sachse

**Affiliations:** ^1^ Structural and Computational Biology Unit European Molecular Biology Laboratory (EMBL) Heidelberg Germany; ^2^ Faculty of Biosciences EMBL and Heidelberg University Heidelberg Germany; ^3^ Ernst‐Ruska Centre for Microscopy and Spectroscopy with Electrons 3/Structural Biology Forschungszentrum Jülich Jülich Germany; ^4^ JuStruct: Jülich Center for Structural Biology Forschungszentrum Jülich Jülich Germany

**Keywords:** Caspar carboxylates, cryo‐EM, helical reconstruction, tobacco mosaic virus, virus assembly/disassembly, Microbiology, Virology & Host Pathogen Interaction, Structural Biology

## Abstract

Stable capsid structures of viruses protect viral RNA while they also require controlled disassembly for releasing the viral genome in the host cell. A detailed understanding of viral disassembly processes and the involved structural switches is still lacking. This process has been extensively studied using tobacco mosaic virus (TMV), and carboxylate interactions are assumed to play a critical part in this process. Here, we present two cryo‐EM structures of the helical TMV assembly at 2.0 and 1.9 Å resolution in conditions of high Ca^2+^ concentration at low pH and in water. Based on our atomic models, we identify the conformational details of the disassembly switch mechanism: In high Ca^2+^/acidic pH environment, the virion is stabilized between neighboring subunits through carboxyl groups E95 and E97 in close proximity to a Ca^2+^ binding site that is shared between two subunits. Upon increase in pH and lower Ca^2+^ levels, mutual repulsion of the E95/E97 pair and Ca^2+^ removal destabilize the network of interactions between adjacent subunits at lower radius and release the switch for viral disassembly.

## Introduction

The capsid of RNA viruses provides a container that ensures the protection of the viral genome from degradation in the extracellular environment. The shell of tobacco mosaic virus is particularly thermostable in ambient temperatures and resistant to degradation across a wide range around neutral pH 1. The TMV capsid is primarily composed of a 17 kDa coat protein (CP) that is organized in a helical assembly thereby tightly enclosing the viral RNA of the genome 2, 3, 4, 5. TMV enters the plant cell through mechanical lesions that transiently open the outer membrane 6. The propagation of the virus is then triggered by a controlled opening of the viral capsid inside the host cell in order to facilitate cotranslational virion disassembly by the replisome 7. This requires a molecular mechanism that is capable of sensing the differences between the extracellular and the intracellular medium, followed by destabilization of the assembled virion and subsequent replication in the host cell. A series of plant viruses use Ca^2+^ and pH triggered disassembly 8 and thereby exploit the lower Ca^2+^ and proton concentrations inside the plant cell compared with the extracellular environment 8.

The disassembly behavior of TMV has been biochemically studied in remarkable detail. Based on titration experiments, it was reported that TMV contains groups that titrate with pK_a_ values between 7 and 8 9, leading to the hypothesis that a carboxylate cluster binds protons with high affinity. These putative residues termed “Caspar‐Carboxylates” 9, 10 are thought to drive virus disassembly through mutual repulsion upon entering the intracellular environment. In addition, it has also been demonstrated that TMV binds Ca^2+^ via these residues 11, 12. The CP folds into a four‐helix bundle that is commonly divided into three regions referring to the radial distance from the helical axis 13: lower radius 20–40 Å, middle radius 40–60 Å, and higher radius 60–90 Å. Mutational studies have identified the critical residues 14, 15 involved in the disassembly process: E50 and D77 at middle radius of the cylinder cross section have been hypothesized to be involved in axial carboxylate interactions, whereas E95, E97, and E106 at lower radius mediate lateral carboxylate and possible Ca^2+^ interactions 4. Moreover, it has been shown by single molecule force spectroscopy, that upon Ca^2+^ decrease the 5′ end of the capsid becomes exposed and RNA‐coat protein interactions are weakened at the rest of the virion 7, presumably to advance cotranslational disassembly by the replisome machinery.

Despite the wealth of biochemical and mutational studies, our structural understanding of the conformational switch sensing the environmental changes is still incomplete. Although TMV was subject to a plethora of structural studies 13, 16, 17, 18, resolution of the helical rod was limited to 2.9 Å when determined by early X‐ray fiber diffraction 5 or later to 3.3 Å by electron cryo‐microscopy (cryo‐EM) studies 13, 16 and more recently up to 2.3 Å resolution 19. At these resolutions, the maps were sufficiently clear for the assignment of the architecture of the CP and annotation of bulky side chains, whereas further details regarding the conformation of the implicated side chains remained undetermined. The proposed Caspar carboxylate residues E95, E97, E106, and the calcium binding site are found in a more flexible part of the protein at lower radius with high B‐factors, as it is expected for such a metastable switch. In the absence of RNA within the disk assembly of TMV at higher resolution, the respective residues were not detectable and assumed to be disordered 20. Generally, negatively charged amino acid residues suffer from faster radiation damage when imaged by cryo‐EM 16, 21, which makes them more difficult to model. Therefore, the precise structural details of this intricate viral disassembly switch remain to be elucidated.

In order to address this outstanding fundamental question regarding viral RNA disassembly, we used cryo‐EM including latest developments of high‐resolution imaging, data processing, and map interpretation methods and determined two ~ 2 Å resolution TMV structures. The maps reveal that the metastable switch is based on a Ca^2+^‐sensitive network of carboxylate and iminocarboxylate residues at lower radius, which become destabilized by Ca^2+^ release at higher pHs. The cryo‐EM structures captured TMV at different conformational states of this network and thereby directly reveal the transitional mechanics of the switch driving viral disassembly.

**Table 1:** Model validation statistics

Model quantity	Ca^2+^/acidic pH	Water (Model 1)	Water (Model 2)	Water (Model 3)
Ramachandran outliers	0.00%	0.00%	0.00%	0.00%
Ramachandran favored	97.35%	97.35	96.69%	96.69%
Rotamer outliers	0.00%	0.00%	0.00%	0.00%
Clashscore	1.6	3.6	4.4	4.4
RMS(bonds)	0.0037	0.0051	0.0056	0.0052
RMS(angles)	0.63	0.76	0.82	0.82
C‐beta deviations	0	0	0	1
MolProbity score	1.03	1.28	1.43	1.43
DipCheck chi‐score	−0.59	−0.61	−0.99	−0.67
RSCC(mask)	0.86	0.86	0.86	0.86

Statistics for the refined Ca^2+^/acidic pH atomic model and the three different models from TMV in water.

## Results and Discussion

### Cryo‐EM structures of TMV at 1.9 and 2.0 Å resolution

**Figure 1:**
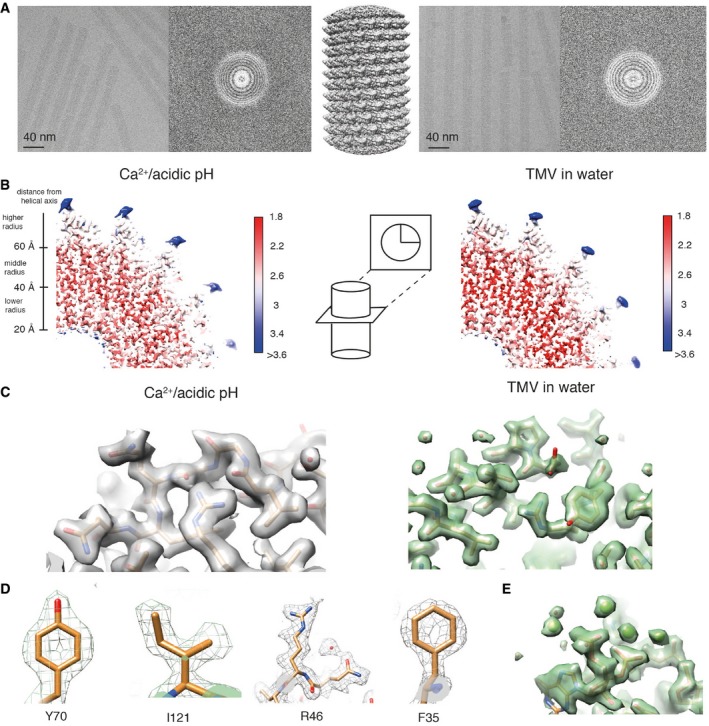
High‐resolution cryo‐EM structures of tobacco mosaic virus (TMV) in conditions of Ca^2+^/acidic pH (gray, left) and water (green, right) Characteristic micrographs for both data sets, respectively.Local resolutions mapped on the respective 3D reconstructions. The center of the coat protein is resolved up to 1.8 Å with some features of approx. 5 Å resolution at the C terminus.Both maps show well‐resolved protein features including water molecules.Side‐chain features of Y70, I121, R46, and F35 residues at Ca^2+^/acidic pH.Snapshot of RNA map features with Mg^2+^ ions in water conditions.

**Figure EV1:**
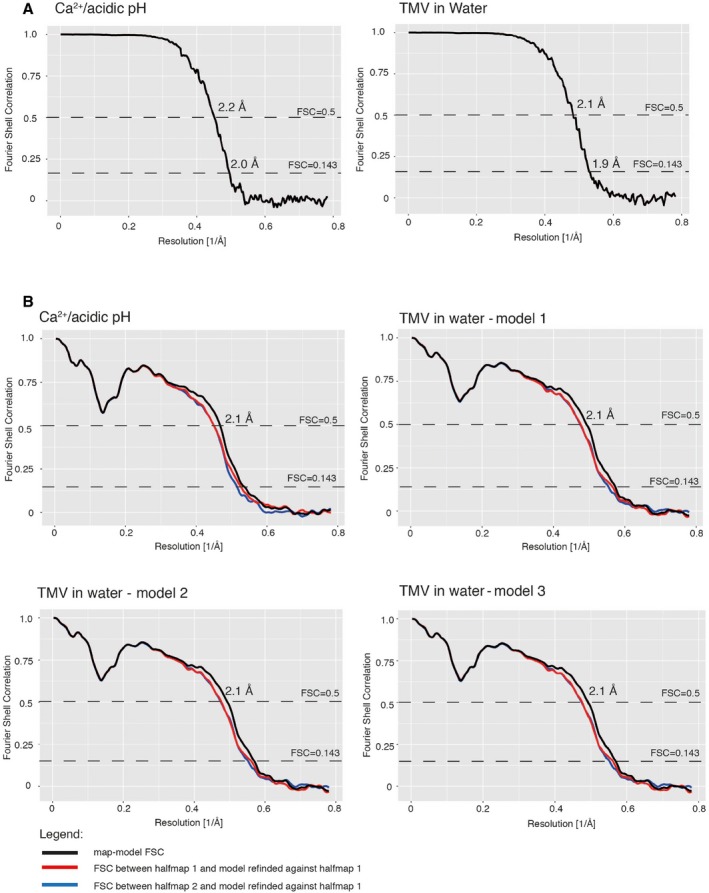
Resolution assessment using Fourier shell correlation (FSC) Comparison of FSC curves between two half‐maps for the Ca^2+^/acidic pH (left) and water structure (right).FSC curves between map and model (black), between half‐map 1 and a perturbed model refined against half‐map 1 (red) as well as between half‐map 2 and a perturbed model refined against half‐map 1 (blue) for the four determined atomic models.

**Figure EV2:**
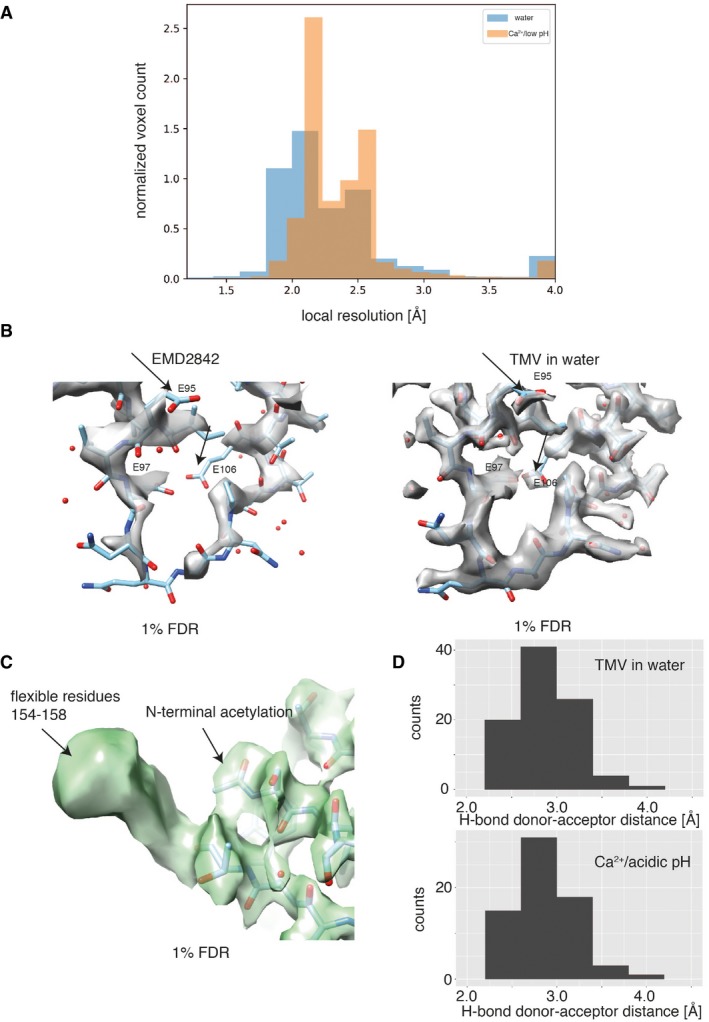
Local resolution assessment of Ca^2+^/acidic pH and water structure Overlay of local resolution histograms computed with BlocRes in the Ca^2+^/acidic pH (orange) and water condition (blue). Resolution of the water map is slightly higher.Map comparison from previous study (left) 16 with this study in water (right) including overlaid current atomic model thresholded at a FDR of 1%. The here‐determined structure shows additional significant and defined map features for the lower radius region.Additional map features for the flexible C terminus and N‐terminal acetylation are significant at an FDR of 1%.Histogram of donor–acceptor distances for the observed hydrogen bonds of modeled water molecules.

**Figure EV3:**
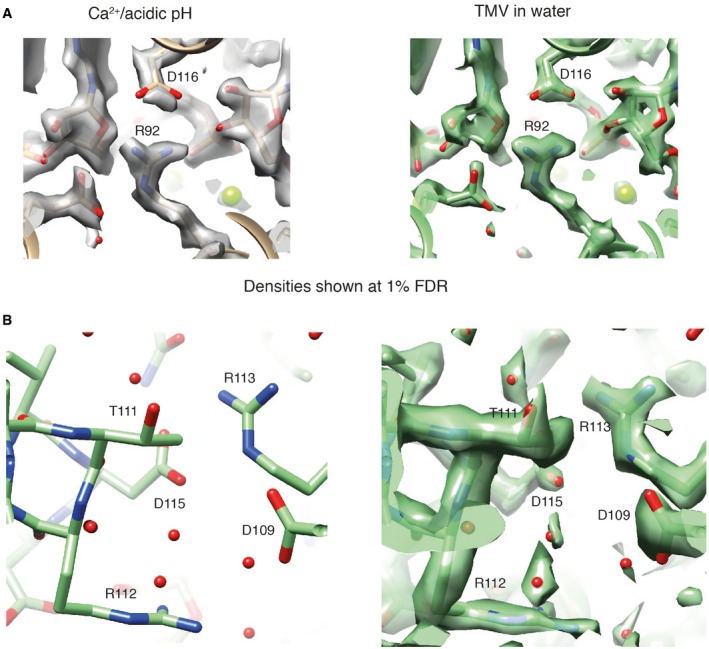
Structural details of Ca^2+^/acidic pH and water states Atomic models shown with the respective maps of TMV in Ca^2+^/acidic pH (left) and water condition (right) at the proposed location of a second Ca^2+^ site in proximity to the RNA 16, 20. No compatible Ca^2+^ ion map features could be detected.Residue D109 and its environment: No obvious interaction with other carboxylates is evident in our structures (left). The same view is shown with corresponding map at 1% FDR (right).

**Figure EV4:**
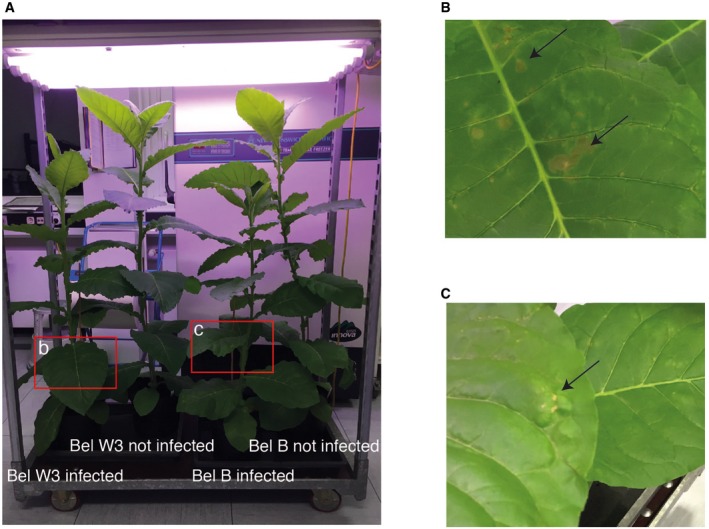
Symptoms of TMV infection on tobacco plants Four tobacco plants from left to right: variant Bel W3 infected, Bel W3 not infected, variant Bel B infected, Bel B not infected. Infected plants are significantly reduced in height in comparison with the non‐infected control plants.Leaf of variant Bel W3 with necrotic lesions (arrows).Leaf of variant Bel B with light green spots (arrow) and bulges.

To elucidate different structural states of the viral assembly, we prepared TMV in two different conditions, first in the presence of 20 mM CaCl_2_ at pH 5.2 (referred to as Ca^2+^/acidic pH) and second in water in the absence of any cations. Both samples were plunge‐frozen and imaged using a 300 kV electron microscope equipped with a GIF Quantum K2 camera (Fig 1A). With the collected micrographs, we determined the 2.0 and 1.9 Å resolution TMV structures in Ca^2+^/acidic pH and water, respectively (Fig EV1A). Both maps show local resolutions up to 1.8 Å for the CP core and ~ 5 Å for the disordered C‐terminal tail (Figs 1B and EV2). Map details agree with the expected high‐resolution features such as defined carbonyl oxygens of the protein backbone. The map can also be used to locate non‐protein components such as water molecules and metal ions. In order to minimize the influence of noise during molecule and ion placements, we used recently developed confidence maps at a 1% false discovery rate (FDR) threshold 22 that is known to suppress noise in comparison with fixed sigma thresholds of EM maps. Using these confidence maps together with expected donor–acceptor hydrogen bond lengths 23 (Fig EV2D), we modeled a total of 92 water molecules for TMV in water and 71 water molecules under Ca^2+^/acidic pH. Due to the proximity to RNA, we modeled 4 Mg^2+^ ions bound to RNA as well as well‐defined side‐chain conformers per CP in both conditions (Fig 1C–E). A critical Ca^2+^ could be located in the Ca^2+^/acidic pH structure whereas no Ca^2+^ binding was observed at the proposed Ca^2+^ site at the RNA 5, 24 in both maps (Fig EV3A). In order to verify the principal biological activity of the imaged virus structures, we demonstrated infectivity of the used virus batch in tobacco plants, which showed typical symptoms like stunted growth and necrotic lesions 35 days post‐infection (Fig EV4).

### Ca^2+^/acidic pH and water structures at lower radius

**Figure 2:**
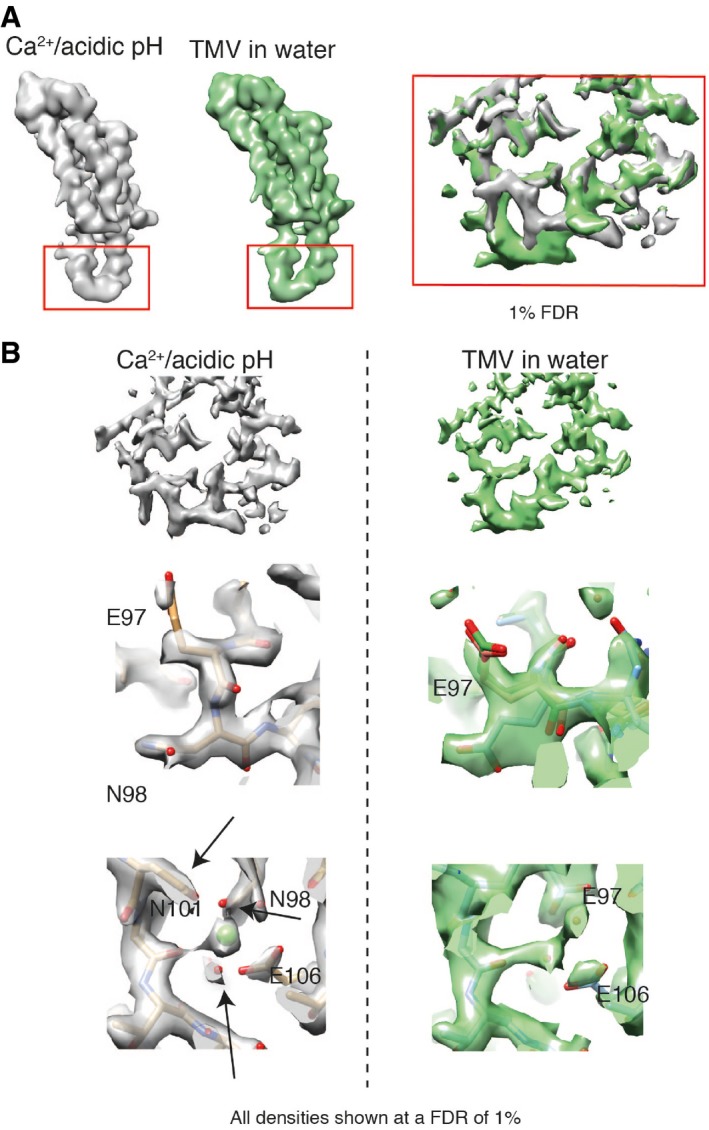
Confidence maps thresholded at 1% FDR from cryo‐EM maps of Ca^2+^/acidic pH and water structure Low‐pass‐filtered monomer map features of Ca^2+^/acidic pH (gray) and water structure (green) with differences at lower radius (left). Zoomed inset (right) with maps displayed at a false discovery rate (FDR) threshold of 1% showing significant differences.Detailed map comparison at lower radius region of Ca^2+^/acidic pH (gray, left column) with the water condition (green, right column). A total of three atomic models (model 1: cyan, model 2: pink, model 3: green) describe the map of TMV in water whereas the Ca^2+^/acidic pH map could be modeled with a single atomic model (center). Located Ca^2+^ ion in the Ca^2+^/acidic pH map with different conformations in the water structure (bottom).

Using our recently developed statistical framework for the annotation of molecular map features 22, i.e., confidence maps that assist in the assignment of atomic models within weaker cryo‐EM map density, we were able to analyze the CP map from Ca^2+^/acidic pH and water samples in a comparative manner (Fig 2A). Although the two determined TMV maps are very similar for most of the CP, the lower radius region differs significantly at an FDR of 1% (Fig 2A right). Comparison of this lower radius map features with recently determined EM map features EMD2842 16 (Fig EV2B) revealed that previous studies only poorly resolved this part of the protein. Detailed comparison of the Ca^2+^/acidic pH and water structure at the lower radius region showed that the protein backbone follows a different path (Fig 2B top). In the determined Ca^2+^/acidic pH structure, we built an atomic model matching the map (Fig 2B left). In the water structure at lower radius, however, we identified three co‐existing models in the residue range 97–100 that describe the map, e.g., the map of the water structure is consistent with multiple conformations of E97 (Fig 2B right).

**Figure EV5:**
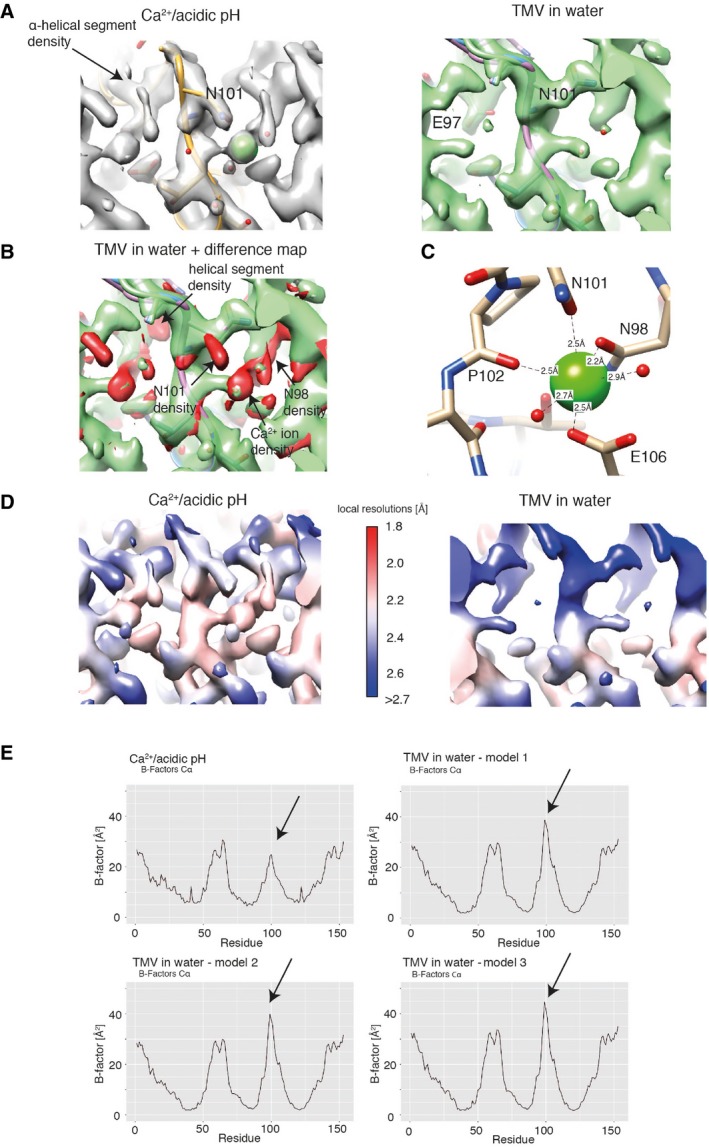
Visualization of structural differences between Ca^2+^/acidic pH and water cryo‐EM maps of determined states TMV at lower radius in the cryo‐EM maps under Ca^2+^/acidic pH condition (left) and of TMV in water (right).The corresponding view of the water cryo‐EM structure (green) together with the difference map (red). Map features corresponding to the Ca^2+^ ion as well as for the coordinating residues and the rearranged helical segment are visible in the difference map.Detailed depiction of the Ca^2+^ ion including coordination distances with neighboring residues.Local resolution plots of the lower radius region for the Ca^2+^/acidic pH map (left) and for TMV in water (right).Plot of Cα B‐factors with corresponding residue number. Plot of four determined models from this study is shown. The peak for residues 90–110 at the lower radius region (highlighted with an arrow) shows lower B‐factors in the Ca^2+^/acidic pH condition. For the remaining residues, the overall profile is very similar.

In order to analyze the two structures in more detail, we refined the atomic coordinates by a common real‐space optimization approach (Table 1) 25. As expected, the structural differences between the Ca^2+^/acidic pH and water structures in the region outside the 90–110 region are very low with RMSDs below 0.5 Å whereas higher between 2.4 and 2.8 Å inside the lower radius region. The multiple models placed for the water structure in the lower radius region (E97–A100) deviate to a smaller but significant extent around 1.0 Å (Table 2). It should be noted that the cryo‐EM density for these three models is not discrete but they are compatible with a flexible ensemble of models describing the continuous density. Closer inspection of the lower radius interface between neighboring subunits reveals additional differences: In the Ca^2+^/acidic pH structure, map features of a bound ion are present and consistent with coordination by E106, N101, N98, and a backbone carbonyl oxygen. In the water structure, however, this complete coordination is missing and asparagines N101 and N98 are facing away from the central map features. In order to confirm the observed differences on the level of the cryo‐EM maps, we compared the respective loop regions by difference mapping (Fig EV5A). The difference map between the two conditions shows the rearrangement of the α‐helical segment including the presence and coordination of the Ca^2+^ ion (Fig EV5B). Annotation of the Ca^2+^ ion is based on high map values at the respective site and octahedral coordination (Fig EV5C). Moreover, local resolutions plots of the cryo‐EM maps justify the placement of side chains and show once more the stabilizing effect of the Ca^2+^/acidic pH condition (Fig EV5D). Therefore, we conclude that under Ca^2+^/acidic pH conditions, this subunit interface is stabilized by a Ca^2+^ ion, whereas in TMV in water the respective site is occupied by water molecules.

**Table 2:** RMSD values for model comparisons

	Water (Model 1)	Water (Model 2)	Water (Model 3)
(a) Main and side‐chain RMSD between models of TMV (90–110)			
Ca^2+^/acidic pH	2.80 Å	2.53 Å	2.38 Å
Water (Model 1)	–	0.94 Å	1.28 Å
Water (Model 2)	–	–	0.75 Å
(b) Main and side‐chain RMSD between models of TMV Δ(90–110)			
Ca^2+^/acidic pH	0.33 Å	0.40 Å	0.40 Å
Water (Model 1)	–	0.24 Å	0.24 Å
Water (Model 2)	–	–	0.01 Å
(c) Main‐chain RMSD between models of TMV (90–110)			
Ca^2+^/acidic pH	2.21 Å	1.93 Å	1.92 Å
Water (Model 1)	–	0.43 Å	0.46 Å
Water (Model 2)	–	–	0.24 Å
(d) Main‐chain RMSD between models TMV Δ(90–110)			
Ca^2+^/acidic pH	0.09 Å	0.09 Å	0.09 Å
Water (Model 1)	–	0.01 Å	0.01 Å
Water (Model 2)	–	–	0.01 Å

RMSD values (main and side chain combined) for residues 90–110 and Δ(90–110) are shown in (a) and (b). The respective main‐chain only RMSD values are shown in (c) and (d).

### Interactions involved in the metastable switch

**Figure 3:**
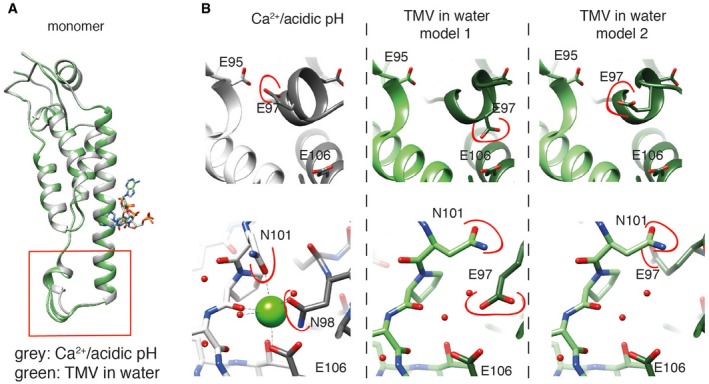
Model comparison of Ca^2+^/acidic pH and water structural states Superposition of the monomer structures with the lower radius region highlighted in the red box. The Ca^2+^/acidic pH state (gray) shows an additional α‐helical segment when compared with the water models (green).Comparison of the E95–E97 interaction (top row) and of the Ca^2+^ binding site in the Ca^2+^/acidic pH model (bottom row). Close proximity of E95 and E97 in the Ca^2+^/acidic pH state, whereas in water, E97 flips toward E106 and is rather flexible. No Ca^2+^ ion and corresponding coordination is evident in the water model. Residues that change conformation are marked in red. Adjacent subunit models are displayed in lighter shade of the main color. Model 3 is not shown due to high structural similarity with model 2 in the displayed region.

**Figure 4:**
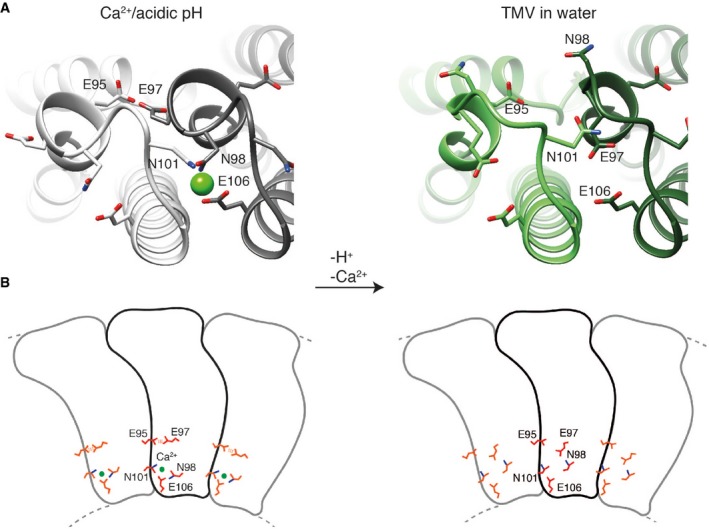
Toward a disassembly mechanism based on the Ca^2+^/acidic pH and water states The close proximity of the E95–E97 interaction and tight coordination of Ca^2+^ in the Ca^2+^/acidic pH state site suggests a mechanism in which, upon pH change and Ca^2+^ removal, repulsive forces between charged carboxylates destabilize the network of interactions at lower radius releasing the switch for viral disassembly.Schematic presentation of coat protein with neighboring subunits including main residues of TMV in the Ca^2+/^acidic pH state (left) and in water (right) responsible for the metastable disassembly switch.

Further comparative analysis of the detailed interactions at lower radius revealed an extended helix of the short α‐helical segment in the Ca^2+^/acidic pH model by the single residue N98 (Fig 3A). Plots of refined atomic B‐factors also show a decrease from 42 to 25 Å^2^ in the lower radius region for the Ca^2+^/acidic pH condition in comparison with water, supporting the notion that Ca^2+^ stabilizes the assembly structure (Fig EV5E). Next, we more closely examined the carboxylate residues previously identified to be critical in the disassembly process (E50, D77, E95, E97, and E106). First at medium radius, E50 and D77 contribute to tight axial carboxylate contacts at a distance of 3.0 Å and showed no differences between the two determined structures. Second at lower radius, we find that glutamates E97 and E95 make up tight inter‐subunit interactions in the Ca^2+^/acidic pH structure with a distance between the carboxylate groups of 2.5 Å (Fig 3B left, top). Although E106 is not found in contact with other carboxylates, E106 is involved in the coordination of Ca^2+^. The respective Ca^2+^ site is shared between the adjacent subunits as it is coordinated by E106 and N98 from one CP monomer and N101 as well as the backbone carbonyl oxygen of P102 from the neighboring monomer (Fig 3B left, bottom). These close inter‐subunit interactions between neighboring CPs add to the stability of the helical assembly. The water structure, however, is lacking the close inter‐subunit carboxylate contacts as E97 shows two different conformations, one facing toward E106 (model 1) and the other toward E95 (model 2) with significantly longer distances of 3.9 and 5.4 Å between carboxylates, respectively (Fig 3B right, top). Residues N101 and N98 also assume different conformations in the water condition (Fig 3B right, bottom), whereas they participate in the coordination of Ca^2+^ in the Ca^2+^/acidic pH condition. We conclude that due to the loss of Ca^2+^ coordination as well as the buildup of carboxylate repulsion at higher pH, the residue network in proximity of E106, N101, and N98 becomes destabilized and changes conformations (Fig 4).

### Toward a structural mechanism of the viral disassembly switch

Based on our two high‐resolution cryo‐EM structures in the presence and absence of Ca^2+^, we propose a detailed structural mechanism of the viral disassembly switch. In the presence of Ca^2+^ at acidic pH, tight inter‐subunit interactions via Ca^2+^ coordination and carboxyl‐carboxyl(ate) interactions between E95 and E97 stabilize the assembly. In these conditions, carboxyl residues are able to bind protons and neutralize their negative charges, which weakens their repulsive force leading to close contact between E95 and E97 (Fig 4). Upon entering the cell, the pH rises and carboxyl groups are deprotonated leading to repulsive forces between them. Putative movement of E97 away from E95 and correlated motion of N98 and N101 destabilize the ion binding site and further promote Ca^2+^ removal in a low Ca^2+^ environment. These concerted conformational rearrangements loosen the stabilizing inter‐subunit interactions and ultimately release the switch to disassemble the virus (Movie EV1). The involvement of additional residues such as N101 and N98 in the coordination of the Ca^2+^ binding site suggests a more intricate conformational network responsible for rearrangements beyond the previously postulated carboxylate pair repulsion driving disassembly, which is corroborated by results from a series of mutation experiments 15. It should be noted that due to the comprehensive nature of conformational changes at the lower radius regions, it is not possible to assign a temporal order to the conformational changes from the two observed structural states of TMV assembly.

Previous studies proposed a critical Ca^2+^ site that interacts with the RNA backbone 5, 24. Such a site could not be located in our two structures (Fig EV3A). In fact, the conformations around the RNA in both our structures resemble what has been referred to as the low Ca^2+^ state 24, with D116, R92, and R90 involved in RNA binding 13 and the direct interaction of R92 and D116. According to our structures, this previously proposed second Ca^2+^ site and the slightly altered conformation at the RNA 5, 24 may not be required for TMV stabilization. In addition, residue D109 thought to be important for disassembly was not found to assume different conformations in the two structures and did not form interactions with one of the before‐mentioned residues (Fig EV3B). To what extent the noted structural differences reflect the different preparation conditions or experimental uncertainties of the previous atomic models is not easy to resolve. Although local resolutions of the here‐determined structures at lower radius drop to ~ 2.5 Å, maps are sufficiently clear to locate all the mentioned side chains with high confidence (Fig 3). In order to confirm that our batch of TMV presents a biologically active virus, we also showed experimentally that our sample is capable of infecting tobacco plants (Fig EV4).

The proposed structural destabilization mechanism offers the possibility of a cooperative disassembly switch between subunits: the removal of a Ca^2+^ from its coordination site at lower radius has an immediate effect on the neighboring Ca^2+^ sites, which are located in close proximity of 10 Å. In the case of the 5′ and 3′ ends of the virion, the end subunits can only weakly interact at the shared Ca^2+^ coordination site in the Ca^2+^/acidic pH condition as they lack the stabilizing neighboring subunit residues. Upon drop of Ca^2+^ concentration, the ends are even further destabilized and more easily accessible to the pulling replisome machinery. This shared Ca^2+^ site provides a direct explanation of preferred capsid opening at the virion ends and cooperative weakening of RNA‐coat protein interactions thus facilitating cotranslational virion disassembly by the replisome 7. Although the lower radius region of the virion is destabilized in low Ca^2+^ and basic environments, we find that the large part of the CP conformation is not affected by these environmental changes. This is an important aspect of the CP plasticity, which only requires a subtle destabilization of the metastable switch to trigger cotranslational disassembly 26 and, at the same time, to be sufficiently stable to allow for re‐assembly of the virion after viral replication 7.

## Material and Methods

### Sample preparation

Tobacco mosaic virus sample was isolated as described in ref. 16 and stored in 0.1 M Tris–HCl pH 7.0, 0.02% NaN_3_ (*w*/*v*) at a concentration of ~ 33 mg/ml at 4°C. A total of 50 μl of virus stock solution was dialyzed for 1 h at room temperature against 50 ml of 0.1 M NaOAc pH 5.2, 20 mM CaCl_2,_ and 50 ml of MilliQ H_2_O, respectively. Before plunge‐freezing, sample concentration was adjusted to 22 and 1.1 mg/ml for the Ca^2+^/acidic pH and the water condition, respectively. A total of 3.6 μl were applied on holey carbon grids (C‐flat 300 mesh R2/2, Protochips) that had been glow‐discharged in an EasyGlow (Pelco) device. Grids were plunge‐frozen in liquid ethane using a Vitrobot Mark IV (Thermo Fisher Scientific) with a blotting time of 2 s at 10°C and 100% humidity.

### Electron microscopy

Data acquisition was performed on a Titan Krios microscope (Thermo Fisher Scientific) operated at 300 kV, through a Gatan Quantum 967 LS energy filter using a 20 eV slit width in zero‐loss mode. The dataset was recorded on a Gatan K2‐Summit direct electron detector operated in super‐resolution mode, at a calibrated magnification of 215,000 (resulting in a super‐resolution pixel size of 0.319 Å on the object scale) with a defocus range of 0.15–0.35 μm. For the TMV in water, a total of 20 frames were recorded in movies of 5 s exposure at a dose rate of ~ 2.6 e^−^/physical pix/s, accumulating a total dose of 30.8 e^−^/Å^2^ at the sample level. For the TMV in Ca^2+^/acidic pH conditions, a total of 40 frames were recorded in movies of 4‐s exposure with a dose rate of ~ 3.7 e^−^/physical pix/s, accumulating a total dose of 41.3 e^−^/Å^2^ at the sample level. For both samples, data collection was performed on a single grid using SerialEM 27.

### Image processing

After visual inspection of the micrographs, 62 images for the TMV in water, and 197 images for the TMV in Ca^2+^/acidic pH conditions_,_ were selected and both datasets were processed in the same way. Movie frames were aligned and dose‐compensated with MotionCor2 28 using patch‐based alignment (5 × 5) followed by 1/2 cropping in the Fourier domain, resulting in 2× lower pixel sampling and a pixel size of 0.638 Å. Contrast transfer function parameters for the micrographs were estimated using Gctf 29. Helix coordinates were determined automatically using MicHelixTrace 30, resulting in ~ 20,000 segments for each sample. Complete 2D and 3D classifications and refinements were performed using RELION implementation of single‐particle based helical reconstruction 31, including per‐particle refinement of CTF parameters, correction of estimated beam tilt and “Bayesian polishing” 32. Helical symmetry parameters were refined to a helical rise/rotation of 1.405 Å/22.036° and 1.406 Å/22.038° for the Ca^2+^/acidic and the water structure, respectively. The reported overall resolutions for TMV of 2.0 Å in Ca^2+^/acidic pH and 1.9 Å in water conditions were calculated using the Fourier shell correlation (FSC) 0.143 criterion. The final maps were corrected for the modulation transfer function of the detector and sharpened by applying a negative B factor that was estimated using automated procedures 33 (−41 Å^2^ for the TMV in water and −42 Å^2^ for the TMV in Ca^2+^/acidic pH conditions). Local resolution maps were calculated with BlocRes 34 at a 0.5 FSC cutoff and the maps were subsequently locally filtered. For each local window, we used a hyperbolic tangent low‐pass‐filter with a fall‐off of 0.1 and cutoff frequency given by the local resolution. To annotate significant molecular map features in the 3D reconstruction and to control false positive voxels, confidence maps using local resolution information were generated 22.

### Atomic model building and refinement

Atomic models were built and refined as 9‐mers in order to account for inter‐subunit interactions. PDB *4udv* 16 was used as starting model and rigid body fitted into the processed maps using *Chimera* 35. Additional H_2_O and Mg^2+^/Ca^2+^ ions were placed in the maps where biochemically appropriate and confirmed using the 1% FDR thresholded maps. Mg^2+^ was placed in proximity of the RNA and justified by the known tendency of RNA to be stabilized by Mg^2+^ ions. The Ca^2+^ ion was identified by the combination of high map values, octahedral coordination, and lower B‐factors in the respective region (Fig EV5), which distinguishes it clearly from water molecules. The Ca^2+^ ion was only found in the cryo‐EM structure with high Ca^2+^ concentrations. Several rounds of real‐space refinement with *phenix.real_space_refine* 36 using electron scattering factors and manual rebuilding with *Coot* 37 were done to obtain the presented models. Refinement was performed using rotamer, Ramachandran, and C‐β restraints in addition to standard restraints of bond lengths, angles, etc. Real‐space refinement was carried out with global minimization and local grid search options activated. Atomic coordinates and B‐factors were refined against the sharpened and locally filtered maps. Residues 154–158 were not modeled as the corresponding map features were not sufficiently well resolved. An additional significant map feature at 1% FDR at the N terminus was modeled as a previously reported N‐terminal acetylation 38. Grouped atomic displacement factors (ADP) were refined with *phenix.real_space_refine*. Validation scores were calculated with *phenix.molprobity* 39, *phenix.em_ringer* 40, and DipCheck 41. To assess overfitting of the refinement, we introduced random coordinate shifts into the final models using the program *phenix.pdbtools* with the shake option and a mean error of 0.5 Å, followed by refinement against the first unfiltered half‐map (half‐map 1) with the same parameters as above. Comparisons of FSC curves of the randomized model refined against half‐map 1 versus half‐map 1 and the FSC curve of the same model map versus half‐map 2 do not indicate overfitting (Fig EV1B). Simulated model maps were calculated with *LocScale* 42.

### Infection of *Nicotiana tabacum* with TMV

The Bel B and Bel W3 variants of *N. tabacum* were chosen for infection experiments as they are known to be sensitive to TMV. Seeds distributed on a soil surface were watered and placed in a greenhouse for germination and cultivation at the following growth conditions: length of day: 14 h, day: 28°C/night: 22°C, relative humidity: 70%. Seedlings were piqued after 16 days, repotted after 31 days for the first time, and repotted after 58 days for the second time. Before infection, the plants reached a height of 65–85 cm. On Day 60, one plant of both variants was infected with the TMV whereas the second plant was cultivated free of virus as a control. They were grown under ambient room temperature conditions and 16 h of neon light. For infection, 25 μl of tobacco mosaic virus stock (33 mg/ml) was diluted into 10 ml PBS pH 7.5 and mixed with 105 mg Silicon carbide (SiC, 200–450 mesh, Sigma‐Aldrich) in a porcelain mortar. SiC was used as an abrasive to cause small wounds and lesions supporting virus entry 43, 44. The pestle was dipped into the virus/SiC suspension and rubbed gently onto the top surface of each plant leaf 45, 46. Ten days after the infection event, first symptoms of TMV replication were visible 47. The variety Bel B developed deformed leaves, yellowish spots, and new leaves were unusually light green and showed stunted growth. The variety Bel W3 showed lesions, necrotic spots on the leaves, and stunted growth. After 35 days of infection, the plants possessed heights of 100 and 124 cm of Bel B and of Bel W3, respectively, whereas the corresponding non‐infected control plants were of 168 and 167 cm heights.

### Figure preparation

FSC and ADP graphics were visualized with ggplot2 in R 48, 49. Chimera 35 was used for the figure preparation of the molecular maps and atomic models and for preparation of the movie.

## Author contributions

FW, MB, and CS designed research. FW prepared cryo‐samples, acquired data, and computed 3D reconstructions. MB built and interpreted atomic models. IvdH performed tobacco infection experiments. MB, FW, and CS wrote the article with input from IvdH.

## Conflict of interest

The authors declare that they have no conflict of interest.

## Supporting information



Expanded View Figures PDF

Movie EV1

Review Process File

## Data Availability

The accession numbers for the Ca^2+^(acidic)/water cryo‐EM maps, and corresponding atomic coordinate models are EMD‐10130/EMD‐10129 (EMDB, www.ebi.ac.uk/pdbe/emdb) and PDB ID 6SAG/PDB ID 6SAE (PDB, www.ebi.ac.uk/pdbe), respectively. The two TMV micrograph sets of the Ca^2+^/acidic and water condition have been deposited to EMPIAR databank and have been assigned accession ID 10306 and 10305, respectively.
