# Supplementary material for: Elucidation of the viral disassembly switch of tobacco mosaic virus
Source: EMBO Rep. 2019 Sep 19;20(11):e48451. doi: 10.15252/embr.201948451 (PMC6831999; doi:10.15252/embr.201948451)
Supplement: Supplementary file 1 — Expanded View Figures PDF [file EMBR-20-e48451-s001.pdf]

Expanded View Figures

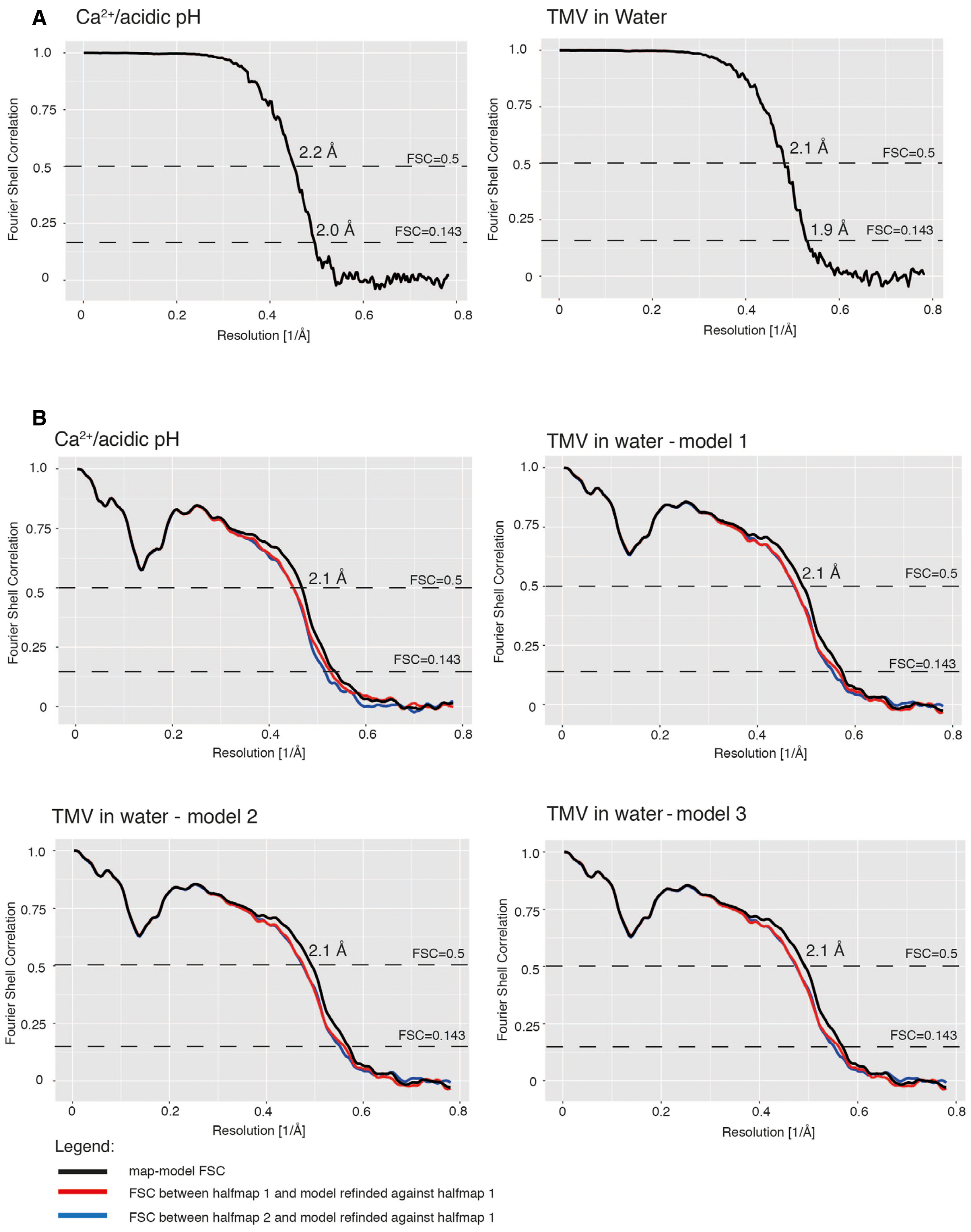

Figure EV1. Resolution assessment using Fourier shell correlation (FSC).

A Comparison of FSC curves between two half-maps for the Ca^{2+}/acidic pH (left) and water structure (right).

B FSC curves between map and model (black), between half-map 1 and a perturbed model refined against half-map 1 (red) as well as between half-map 2 and a perturbed model refined against half-map 1 (blue) for the four determined atomic models.

© 2019 The Authors

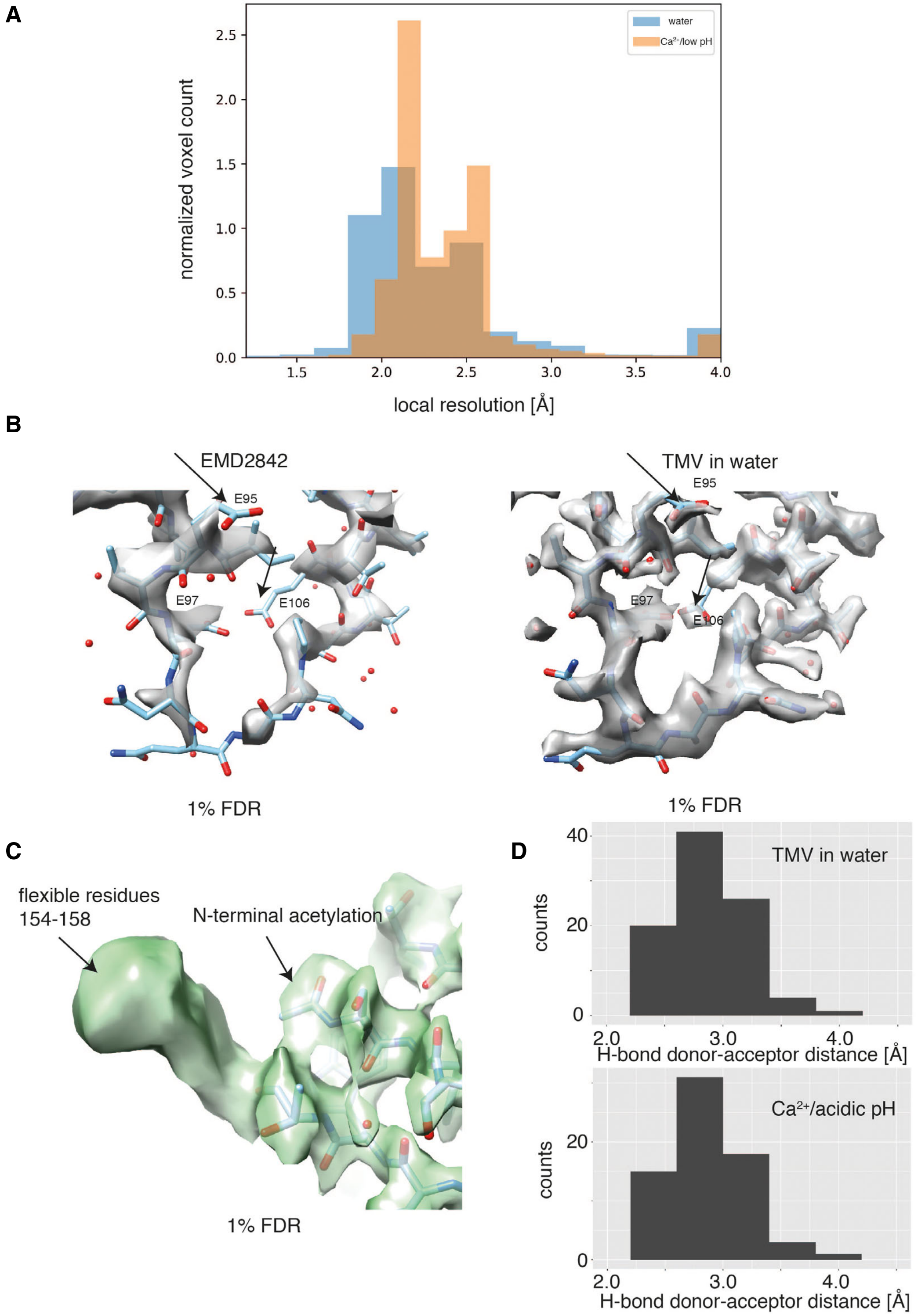

Figure EV2. Local resolution assessment of Ca^{2+}/acidic pH and water structure.

A Overlay of local resolution histograms computed with BlocRes in the Ca^{2+}/acidic pH (orange) and water condition (blue). Resolution of the water map is slightly higher.

B Map comparison from previous study (left) [16] with this study in water (right) including overlaid current atomic model thresholded at a FDR of 1%. The here-determined structure shows additional significant and defined map features for the lower radius region.

C Additional map features for the flexible C terminus and N-terminal acetylation are significant at an FDR of 1%.

D Histogram of donor–acceptor distances for the observed hydrogen bonds of modeled water molecules.

© 2019 The Authors

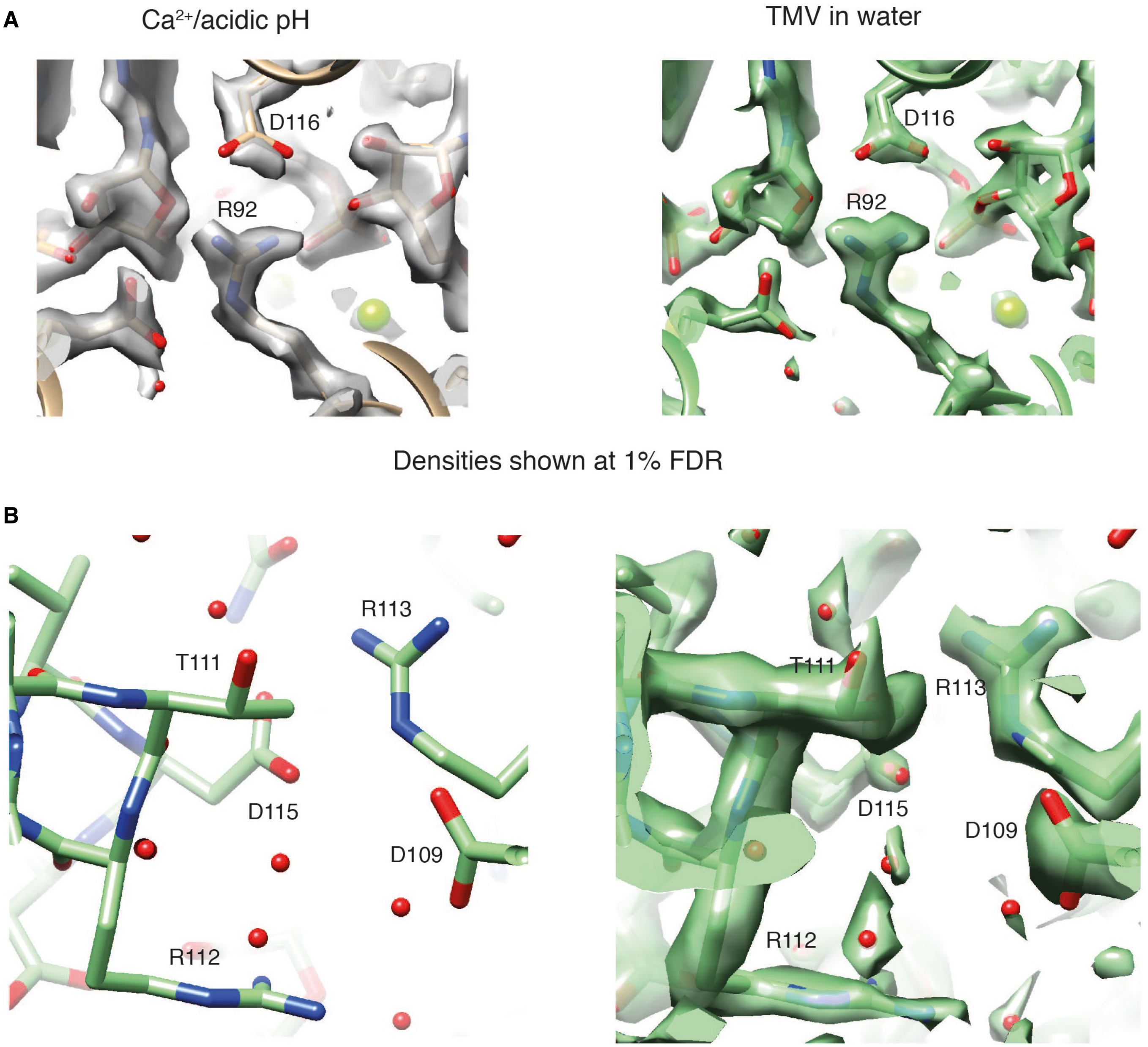

Figure EV3. Structural details of Ca^{2+}/acidic pH and water states.

A Atomic models shown with the respective maps of TMV in Ca^{2+}/acidic pH (left) and water condition (right) at the proposed location of a second Ca^{2+} site in proximity to the RNA [16,20]. No compatible Ca^{2+} ion map features could be detected.

B Residue D109 and its environment: No obvious interaction with other carboxylates is evident in our structures (left). The same view is shown with corresponding map at 1% FDR (right).

 © 2019 The Authors

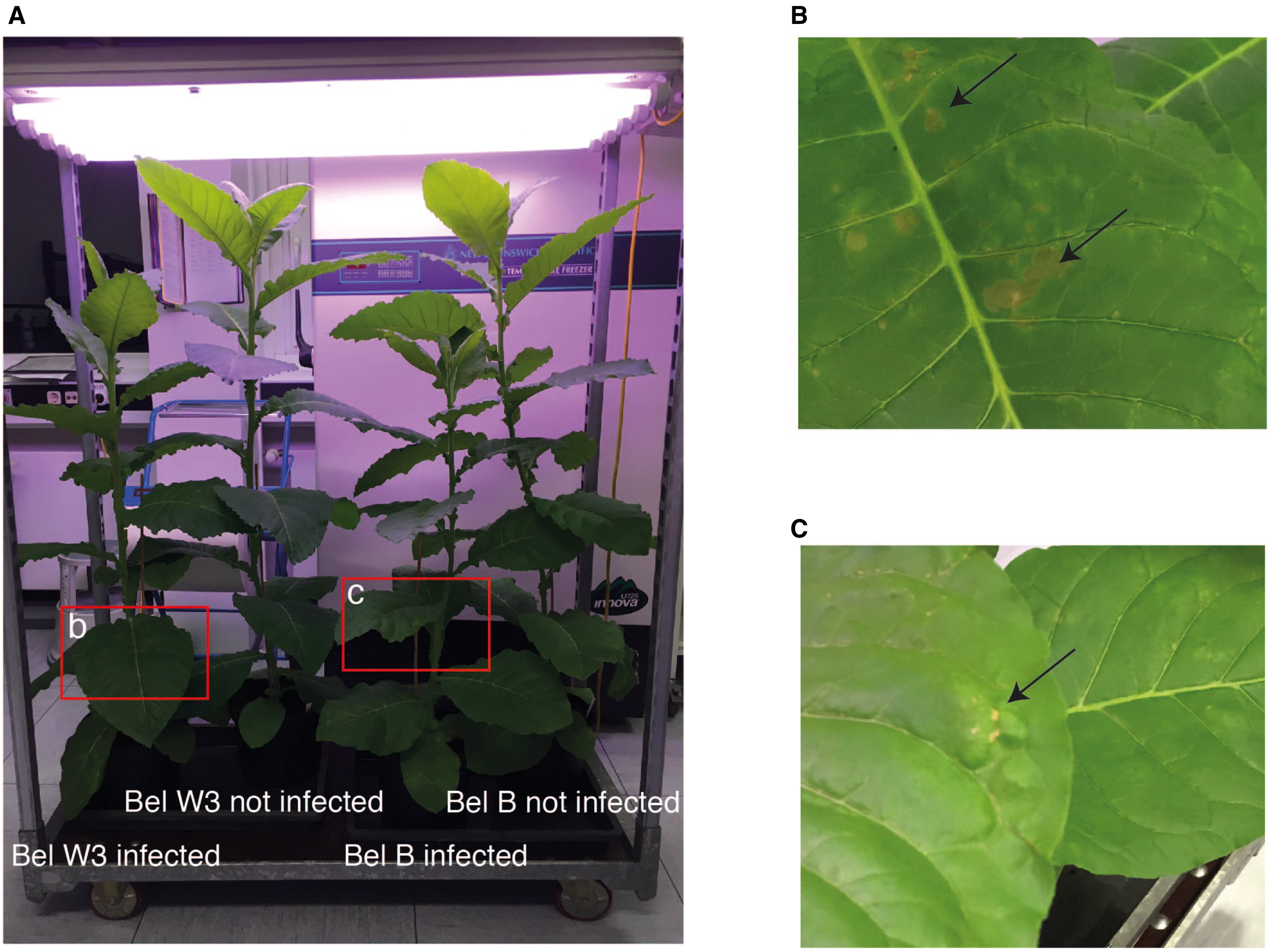

Figure EV4. Symptoms of TMV infection on tobacco plants.

A Four tobacco plants from left to right: variant Bel W3 infected, Bel W3 not infected, variant Bel B infected, Bel B not infected. Infected plants are significantly reduced in height in comparison with the non-infected control plants.

B Leaf of variant Bel W3 with necrotic lesions (arrows).

C Leaf of variant Bel B with light green spots (arrow) and bulges.

© 2019 The Authors

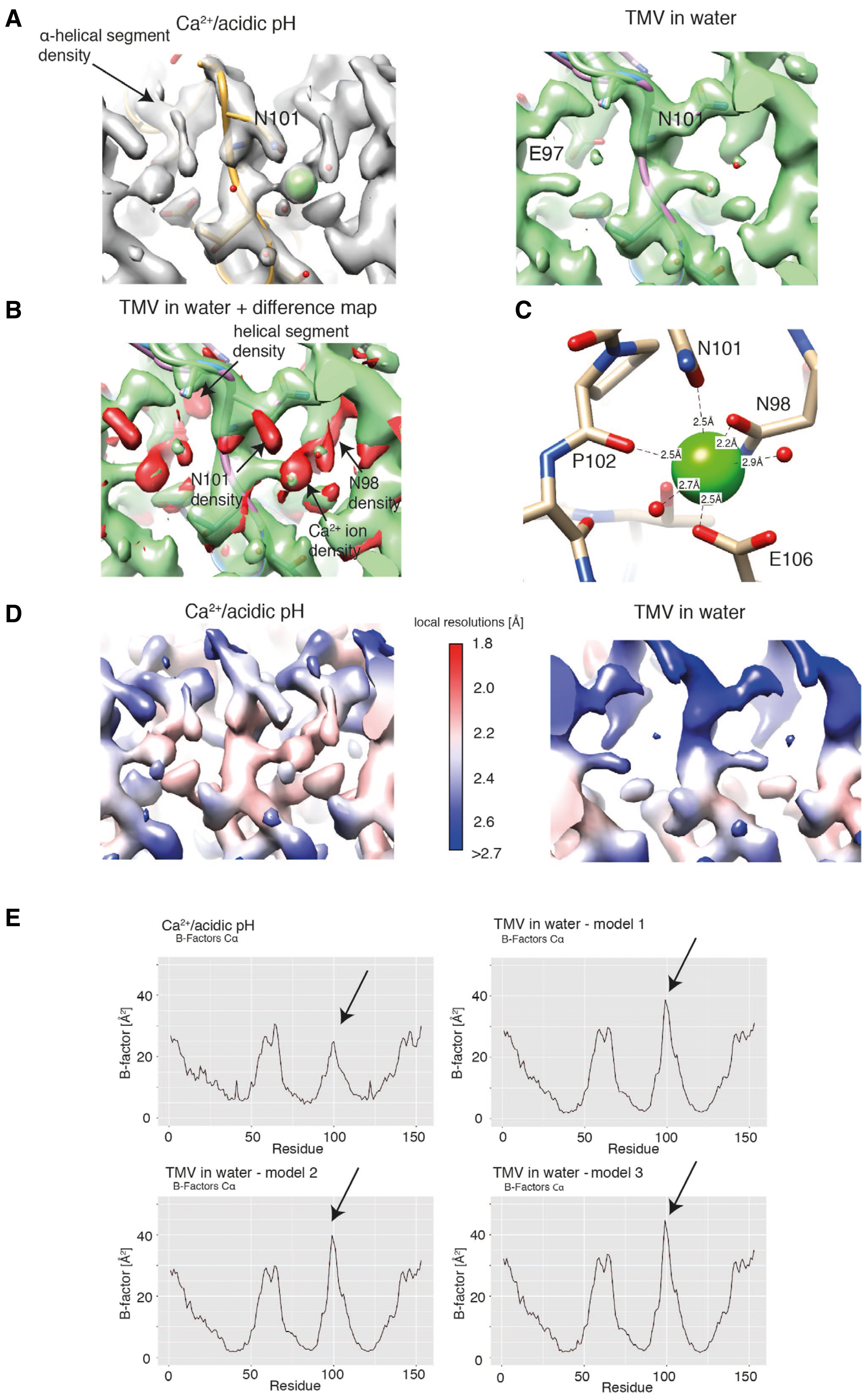

Figure EV5. Visualization of structural differences between Ca^{2+}/acidic pH and water cryo-EM maps of determined states.

A TMV at lower radius in the cryo-EM maps under Ca^{2+}/acidic pH condition (left) and of TMV in water (right).

B The corresponding view of the water cryo-EM structure (green) together with the difference map (red). Map features corresponding to the Ca^{2+} ion as well as for the coordinating residues and the rearranged helical segment are visible in the difference map.

C Detailed depiction of the Ca^{2+} ion including coordination distances with neighboring residues.

D Local resolution plots of the lower radius region for the Ca^{2+}/acidic pH map (left) and for TMV in water (right).

E Plot of Cα B-factors with corresponding residue number. Plot of four determined models from this study is shown. The peak for residues 90–110 at the lower radius region (highlighted with an arrow) shows lower B-factors in the Ca^{2+}/acidic pH condition. For the remaining residues, the overall profile is very similar.

 © 2019 The Authors
