# Supplementary material for: Elucidation of the viral disassembly switch of tobacco mosaic virus
Source: EMBO Rep. 2019 Sep 19;20(11):e48451. doi: 10.15252/embr.201948451 (PMC6831999; doi:10.15252/embr.201948451)
Supplement: Supplementary file 2 — Movie EV1 [file EMBR-20-e48451-s002.zip › MovieEV1/MovieEV1.docx]

**Movie EV1. Structural transition of the determined Ca^2+^/acidic pH and water state.**

Movie shows Ca^2+^/acidic pH structure of the helical TMV assembly and subsequently zooms in the Ca^2+^ site with all residues involved in the switch mechanism highlighted in atom display. The structural transition was interpolated between the Ca^2+^/acidic pH model and model 1 of the water state. Model 1 showed the most different conformation compared with the Ca^2+^/acidic pH structure. Subsequently, all 3 models of the water structure are shown.
